# Unexpected infection outcomes of China-origin H7N9 low pathogenicity avian influenza virus in turkeys

**DOI:** 10.1038/s41598-018-25062-y

**Published:** 2018-05-09

**Authors:** Marek J. Slomka, Amanda H. Seekings, Sahar Mahmood, Saumya Thomas, Anita Puranik, Samantha Watson, Alexander M. P. Byrne, Daniel Hicks, Alejandro Nunez, Ian H. Brown, Sharon M. Brookes

**Affiliations:** 1 Avian Virology and Mammalian Influenza Research, Virology Department, Animal and Plant Health Agency (APHA-Weybridge), Woodham Lane, Addlestone, Surrey, KT15 3NB United Kingdom; 2 Animal Services Unit, Animal and Plant Health Agency (APHA-Weybridge), Woodham Lane, Addlestone, Surrey, KT15 3NB United Kingdom; 3 Pathology Department, Animal and Plant Health Agency (APHA-Weybridge), Woodham Lane, Addlestone, Surrey, KT15 3NB United Kingdom

## Abstract

The China-origin H7N9 low pathogenicity avian influenza virus (LPAIV) emerged as a zoonotic threat in 2013 where it continues to circulate in live poultry markets. Absence of overt clinical signs in poultry is a typical LPAIV infection outcome, and has contributed to its insidious maintenance in China. This study is the first description of H7N9 LPAIV (A/Anhui/1/13) infection in turkeys, with efficient transmission to two additional rounds of introduced contact turkeys which all became infected during cohousing. Surprisingly, mortality was observed in six of eight (75%) second-round contact turkeys which is unusual for LPAIV infection, with unexpected systemic dissemination to many organs beyond the respiratory and enteric tracts, but interestingly no accompanying mutation to highly pathogenic AIV. The intravenous pathogenicity index score for a turkey-derived isolate (0.39) affirmed the LPAIV phenotype. However, the amino acid change L235Q in the haemagglutinin gene occurred in directly-infected turkeys and transmitted to the contacts, including those that died and the two which resolved infection to survive to the end of the study. This polymorphism was indicative of a reversion from mammalian to avian adaptation for the H7N9 virus. This study underlined a new risk to poultry in the event of H7N9 spread beyond China.

## Introduction

A novel H7N9 influenza A virus (IAV) emerged from poultry as a zoonotic infection in China in 2013^1^. A series of annual winter epidemics of respiratory infection in humans have occurred^2^, with 1589 confirmed cases, including 616 deaths, by 20th September 2017^3^, the majority of which have been associated with poultry contact at live poultry markets (LPMs)^1,4^. Widespread dissemination of H7N9 within poultry in China is attributed to it being a low pathogenicity avian influenza virus (LPAIV) with no obvious clinical signs in birds, especially in the chicken which is assumed to be the main maintenance host^5,6^.

Genetic polymorphisms in this H7N9 LPAIV include Q235L (complete gene numbering) in the haemagglutinin (HA) gene of many human isolates which reflects mammalian adaptation. This residue is also identified as position 217 in the H7 HA1 mature peptide which corresponds to position 226 in the H3 numbering convention^7^. The E627K (PB2 gene) polymorphism has been reported in in many human isolates^8^ with continuing evolution evident at the full-genome level in both human and poultry isolates^1,9–11^. The H7N9 internal genes originated from endemic Chinese H9N2 viruses via reassortment^1,9^. However, despite its widespread circulation in poultry in several Chinese provinces, the emergence of a highly pathogenic (HP)AIV cleavage site (CS) sequence was reported relatively recently during winter 2016–2017^12,13^.

Several published *in vivo* poultry studies of China-origin H7N9 LPAIV have included chickens^14–19^, using mainly the prototype human-origin A/Anhui/1/13 H7N9 LPAIV isolate^7^ for infection, with absence of overt clinical signs reflecting the field situation in China. Inclusion of contact chickens enabled the transmission frequency within this species to be assessed, with outcomes ranging from highly efficient or intermediate infection of contacts^14,15,18^ to nil transmission^16,19^. However, a proportion of H7N9 LPAIV human isolates and LPM isolates from avian and environmental specimens possess the Q235 polymorphism which occurs in avian-origin IAVs^10,14^. Although H7N9 LPAIV has been isolated occasionally from domestic ducks in Chinese LPMs^10,14^, experimental investigation has shown that ducks and geese are infected less efficiently *in vivo* with infrequent or poor transmission^17,19^. Turkeys, however, are an insignificant poultry species in China, which contrasts with the substantial commercial turkey sector in Europe and North America^20^. Given that any dissemination to new geographic regions might result in infection of non-chicken hosts^21,22^, this study compared the *in vivo* susceptibility of chickens and turkeys to China-origin H7N9 LPAIV, and evaluated its pathogenicity and transmission within turkeys.

## Materials and Methods

### Viruses

A/Anhui/1/13 H7N9 LPAIV was received from the National Institute for Medical Research, UK, and had been previously passaged through three rounds (egg passage (EP)3) using 9–11 days-old specific pathogen free (SPF) embryonated chickens’ eggs^23^. Three further passages at APHA produced the EP6 stock which served as the inoculum in the *in vivo* experiments, and henceforth will be referred to as H7N9 “wild type” (wt). The EP6 H7N9 was full-genome sequenced and confirmed as identical to the original GenBank sequence submission (accession numbers: CY187618-CY187625) except for an amino acid change in the HA, namely N141D (complete gene numbering). Virus isolation (VI) from clinical specimens derived from the *in vivo* experiments in this study was also done in chicken eggs and 12-days-old embryonated turkey eggs^23^. The EP6 stock and H7N9 LPAIV isolates were titrated in chicken eggs to determine the 50% egg infectious dose (EID_50_)^24^.

### Ethics statement and containment work

All animal experiments and procedures required approval from the local APHA Animal Welfare and Ethical Review Body to comply with the relevant European and UK legislation^25^ and were carried-out in accord with the Home Office (UK) Project Licence 70/8332. Any infected poultry which began to display severe clinical signs were euthanised and were recorded as a mortality. UK regulations categorise the H7N9 LPAIV as a SAPO 4 and advisory ACDP 3 pathogen because it is a notifiable animal disease agent^26^ and presents a zoonotic risk^27^. All laboratory and containment work with H7N9 specimens, including infected poultry, was done in licenced BSL 3 facilities^27,28^.

### Experimental design

**Figure 1:**
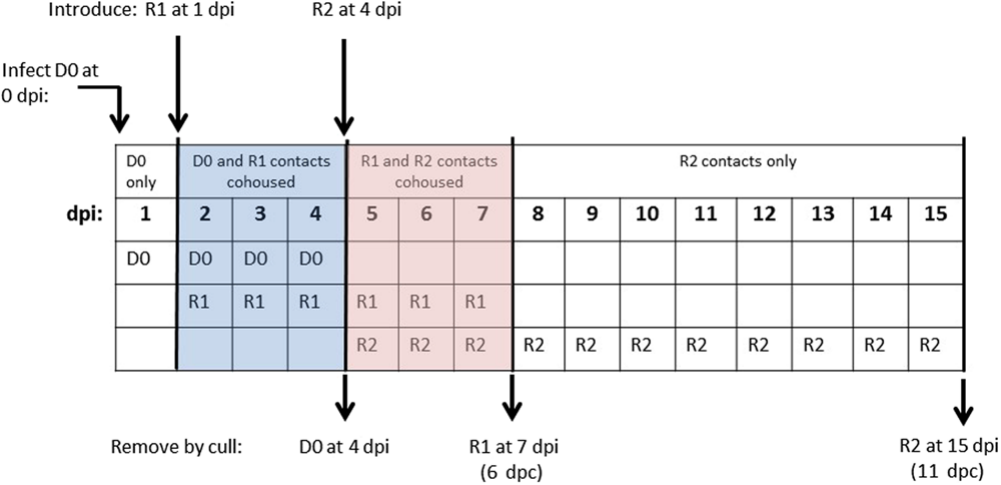
Schematic showing the plan for *in vivo* transmission of H7N9 LPAIV. Direct-inoculation of D0 (“donor”) birds followed by introduction of two rounds of R1 and R2 (“recipient”) contact birds into each pen, including the planned removal (by culling) of D0 and R1 birds at 4 and 7 dpi respectively. Cohousing periods (3-days) for D0/R1 and R1/R2 birds are indicated by blue and pink shading respectively, with R2 birds intended to remain (unshaded days) to the conclusion of the study at 15 dpi. See Results for subsequent differences from this plan.

Prior to infection, birds were swabbed and bled for M-gene RRT-PCR and serological testing to exclude previous AIV exposure. Sixteen SPF chickens (White Leghorn, Valo) and 48 turkeys (Commercial White, high health status, Aviagen) were used to attempt a transmission chain by direct-inoculation followed by contact exposure. All birds were 3-weeks-age, with six chickens and 18 turkeys being directly-inoculated (100 µl per bird, via the ocular-nasal route) and referred to as “donor” (D0) birds in that they served to attempt to transmit infection to contact birds. Contacts are referred to as “recipients”, abbreviated as R1 birds for “first contacts” which were introduced for cohousing with D0 birds at 1 day post-infection (dpi) (Fig. 1). Cohousing investigated transmission of infection via the natural contact route and was attempted in four experimental units, summarised as follows:

- Chicken (D0) to chicken (R1) transmission, with H7N9 wt used to directly-inoculate one group of D0 chickens housed in a single pen using a single dose of 8log_10_ EID_50_ per chicken.
- Turkey (D0) to turkey (R1) transmission, with three different doses of H7N9 wt used to directly-inoculate three separate groups of D0 turkeys housed in separate pens, namely 8, 6 and 4log_10_ EID_50_ per turkey (Fig. 1).

The four experimental units were divided for housing into four Perspex pens (120 cm × 120 cm (total floor space 1.44 m^2^) x 60 cm height) which provided housing density in accord with UK animal welfare recommendations^29^. Details of infection and cohousing which included attempts to extend the transmission chain beyond the R1 birds are outlined for both species:

#### Chicken to chicken transmission, with attempted further transmission to turkeys

An ocular/nasal dose of 8log_10_ EID_50_^18^ of H7N9 wt was directly administered to six D0 chickens. At 1 dpi an equal number of six R1 chickens were introduced into the pen for cohousing for three days when the six D0 chickens were withdrawn at 4 dpi (3 days post-contact (dpc)). Replacement of the six D0 chickens with six R2 (“second contacts”) turkeys at 4 dpi was informed by the transmission findings observed at the same time in the turkey-to-turkey transmission groups (described below): The six R1 chickens were withdrawn at 7 dpi (6 dpc), with the four R2 turkeys remaining to the conclusion of the study at 15 dpi, i.e. 11 dpc.

#### Turkey to turkey transmission

An ocular/nasal dose of 8log_10_ EID_50_ of H7N9 wt was directly administered to six D0 turkeys in a single pen, with two additional turkey pens housing six D0 turkeys in each for direct-inoculation at 6log_10_ EID_50_ and 4log_10_ EID_50_. At 1 dpi an equal number of R1 turkeys were introduced to each of the three D0 turkey groups (Fig. 1). For the two pens which contained D0 turkeys directly-inoculated with the two highest H7N9 doses, cohousing continued for three days before the six D0 turkeys were withdrawn at 4 dpi from each pen, culled and replaced with four R2 turkeys to extend the transmission chain, i.e. from R1 to R2 turkeys (Fig. 1). It was planned to withdraw and cull the R1 turkeys from the three pens at 7 dpi (6 dpc), and to allow the R2 turkeys in these two pens to remain to the conclusion of the study at 15 dpi (11 dpc). In the pen containing six D0 turkeys infected with the lowest H7N9 dose (4log_10_ EID_50_ per turkey), six R1 turkeys were introduced at 1 dpi, with withdrawal and culling at 4 dpi and 7 dpi (6 dpc) respectively with no R2 turkeys introduced due to the inefficient acquisition of infection by the R1 turkeys.

### Collection of clinical specimens from chickens and turkeys

Buccal and cloacal swabs were collected daily from all birds and stored in 1 ml of brain heart infusion broth containing antibiotics, namely 1000 IU penicillin G, 10 mg/ml amphotericin B and 1 mg/ml gentamicin (BHIB). Organs (see Results) were collected from birds which were either culled according to the experimental plan (Fig. 1) or died, and were cut for 10% (w/v) suspension in BHIB^30^. Blood was collected for serum separation from birds at cull or which survived to the conclusion of the experiment at 15 dpi.

### RNA extraction and AIV reverse transcription Real-Time PCR (RRT-PCR)

RNA was extracted from BHIB swab fluids and tissue supernatants by robotic and manual methods respectively^30^. All extracted RNA specimens were tested by the M-gene RRT-PCR, whereby 2 µl RNA was added to a final 25 µl reaction volume containing 12.5 µl (×2) QuantiFast Probe RT-PCR Master Mix (Qiagen), 0.5 µl (×50) ROX dye solution and 0.25 µl QuantiFast RT Mix (from same Qiagen kit), the primers and probe^31^ at final concentrations of 0.6 µM and 0.2 µM respectively. The proprietary lock nucleic acid probe is available from the Roche universal probe library (UPL) 104^31^ with the sequence 5′FAM-CTGGGCAC-BHQ1–3′ (Roche). Thermocycling was performed using an Agilent Mx3000 instrument (Stratagene) as described^31^ with the RT inactivation at 95 °C shortened to 5 minutes and the number of PCR cycles reduced to 40. Ct values < 36 were considered as AIV positive, while those in the range Ct 36.01–39.99 were sub-threshold and Ct 40 was negative. A ten-fold dilution series of titrated EP6 RNA was used to construct a standard curve using MxPro software (Stratagene) to determine PCR efficiency which assured optimal assay performance for quantitative interpretation^30^. Ct values obtained from clinical specimens were converted to relative equivalent units (REUs) by plotting against the EP6 pre-extraction EID_50_/ml values on the standard curve^32^, and for a series of RRT-PCR experiments the average REU value which corresponded to Ct 36 was used to establish the threshold value for the shedding graphs. Infected individual birds were identified by (i) any instance of positive shedding and (ii) sub-threshold shedding from either tract detected on two successive days.

### Post-mortem tissue investigation by immunohistochemistry

Organ samples (Table 1) were fixed in 10% (v/v) buffered formalin for IAV-specific immunohistochemical (IHC) staining by using an anti-nucleoprotein (NP) monoclonal antibody^32^. The frequency of virus-specific staining was assessed semi-quantitatively by examining multiple fields of view for each thin section and was scored as: +uncommon; ++few; +++moderate; ++++abundant.

**Table 1:** Detection of IAV NP antigen in organs by immunohistochemistry.

Species and dose administered to donor (D0) birds	Chickens 8 log_10_ EID_50_				Turkeys 8 log_10_ EID_50_								Turkeys 6 log_10_ EID_50_					Turkeys 4 log_10_ EID_50_	
Directly-infected or contact	D0	D0	R1	R1	D0	D0	R1	R1	R1	R2	R2	R2	D0	D0	R2	R2	R2	D0	D0
Dpi (dpc): all culled (apparently healthy), except E and FD as indicated	4	4	7 (6)	7 (6)	4	4	7 (6)	7 (6)	7 (6) FD	8 (4) FD	11 (7) FD	12 (8) FD	4	4	11 (7) E	8 (4) FD	10 (6) E	4	4
Bird ID #:	1	3	11	12	1	3	11	12	9	37	38	39	13	15	141	143	144	25	28
L235Q polymorphism in HA gene	Q235	L235	ND	Q235	Q235	Q235	Q235	Q235	Q235	Q235	Q235	Q235	L235	L235	Q235	Q235	Q235	ND	ND
Organs																			
Nasal cavity (turbinates) **	−	+	−	−	+++	+++	+++	+++	++	++	+	−	+++	+++	++	++	+++	−	++
Airways**	−	−	−	−	+	++	+++	++	++	+++	+++	−	+++	−	++	+++	++	−	−
Lung**	−	−	−	−	++	+++	++	+	++	++	+	+	+++	−	+	+++	++	−	−
Air Sacs**	−	−	−	−	+++	+++	++	++	+++	+++	−	+	+++	−	−	+++	+++	−	−
Proventriculus**	−	−	−	−	++	++	−	N/a	++	−	−	−	−	−	+++	−	−	−	−
Caecal Tonsil**	+	−	−	−	+	+	−	+	−	−	−	−	−	−	−	−	−	−	−
Intestine (enterocytes)	−	−	−	−	++	++	−	−	−	−	−	−	−	−	−	−	−	−	−
Pancreas**	−	−	−	−	++++	++++	++++	++++	++++	++++	++++	+++	−	−	++++	++++	++++	−	−
Spleen**	−	−	−	N/a	+++	+++	−	−	+	−	N/a	−	−	−	+	N/a	++	−	−
Bursa**	−	−	−	−	+	+	++	++	−	++	+++	++	−	−	+++	−	++	−	−
Thymus*	−	−	−	−	+	+	−	−	+	−	−	−	−	−	−	−	−	−	−
Skin*	−	−	−	−	+	+	+	+	++	−	−	−	−	−	−	+	−	−	−
Feather follicles*	−	−	−	−	+	+	−	−	−	−	−	−	−	−	−	−	−	−	−
Heart**	−	−	−	−	+++	++	++	−	+++	+	+	−	−	−	+	+	++	−	−
Skeletal muscle*	−	−	−	−	++	++	−	−	−	−	−	−	−	−	−	−	+	−	−
Brain*	−	−	−	−	+	+	−	−	+	−	−	−	−	−	+	−	−	−	−
Kidney**	−	−	−	−	++	++	+++	−	++	+++	+++	+++	−	−	++	+++	+++	−	−
Liver**	−	−	−	−	++	++	++	−	++	+		−	−	−	−	−	+	−	−
Gonads*	−	−	−	−	+	+	−	++	+	−	N/a	−	−	−	−	+++	+++	N/a	N/a

Four experimental groups indicated in the header, divided by D0 species and direct dose of H7N9wt (log_10_ EID_50_). E = euthanised, FD = found dead. L235Q polymorphisms indicated for individual birds at time of cull or death. * Detected only in endothelial cells; ** Detected in endothelial and parenchymal cells. N/a: not available; ND not done. Additional infected R2 turkeys # 40 and # 142 (see Results) survived to be culled at the end of the study (11 dpc). No detectable IAV-specific NP antigen was detected in these two survivors by IHC.

### Full-genome sequencing and cycle-sequencing of selected regions in the HA gene

For H7N9 isolates grown in chicken and turkey eggs, RNA was extracted as described^30^ but with the carrier RNA excluded, and full-genome sequences obtained by Next Generation Sequencing (NGS) as previously^33^. For molecular pathotyping, RNA was directly extracted from clinical specimens but with the carrier RNA included, followed by GK7.3/GK7.4^34^ amplification across the HA gene CS sequence as described^30^. To investigate the L235Q mammalian/avian adaptation polymorphism in the HA gene, two primers specific for H7N9 wt were designed which flanked this region:

L235Q fwd: 5′-ATTCCCGCAGATGACTAAG-3′

L235Q rev: 5′-ACTGCCCTGCTATCTATGTT-3′

This L235Q primer pair substituted for the GK7.3/GK7.4 primers in the above conventional RT-PCR with identical thermocycling to produce a 436 bp amplicon. Products were confirmed and purified for cycle-sequencing with the two corresponding RT-PCR primers^30^. Raw sequence data was obtained in both orientations from the HA CS and L235Q conventional (i.e. gel detection) RT-PCRs, and was assembled by the SeqMan II suite (Lasergene bioinformatics package, version 13; DNAstar, Madison, USA) to provide the consensus sequence. Any instances of possible mixed L/Q235 polymorphisms were initially identified by the SeqMan II software interpretation, followed by visual inspection of any dual chromatogram peaks at the same nucleotide position. In instances of uncertainty, cycle sequencing of the purified amplicons was repeated to identify residue 235 as either distinct L or Q amino acids, or as a mixed population of both indicated by X.

### Analysis of H7N9 genetic polymorphisms

In order to assess the possible significance of any H7N9 genetic polymorphisms which emerged following *in vivo* infection of chickens and turkeys, 7792 sequences of the relevant China-origin H7N9 genes were downloaded from the GISAID database (http://platform.gisaid.org/ep/i3) on 24/1/2018 (deposited since 2013) and included 77 sequences from H7N9 HPAIVs deposited since early 2017. The downloaded sequences were aligned along with the corresponding gene sequences from the isolated progeny viruses by using the MAFFT multiple sequence alignment software (version 7)^35^.

### H7- and AIV-specific serology

Sera derived from clotted blood were incubated at 56 °C. Turkey sera (100 µl) were pre-absorbed by adding 20 µl packed chicken red blood cells and incubated for 30 minutes at ambient temperature with occasional agitation. HI testing for HA-specific antibodies in both poultry species used the homologous H7N9 viral antigen titrated to 4 haemagglutination units^23^. Sera were also tested by the multi-species influenza A ELISA (IDEXX, France) and the FluA ELISA (ID Vet, France), both of which are blocking ELISAs that detect antibodies against the IAV type-specific NP antigen. The H7-specific antibody ELISA (version 2, ID Vet) was also used.

### Intravenous pathogenicity index (IVPI)

The IVPI test was performed and scored by standard methods^23^ with egg fluids diluted to a titre of 9log_10_ EID_50_/ml^18^ and 100 µl administered intravenously per four-week-old chicken. If a chicken required euthanasia during the 10-day IVPI determination, it was recorded as having been found dead on the next day.

## Results

### Infection and transmission of H7N9 LPAIV in chickens and turkeys

**Figure 2:**
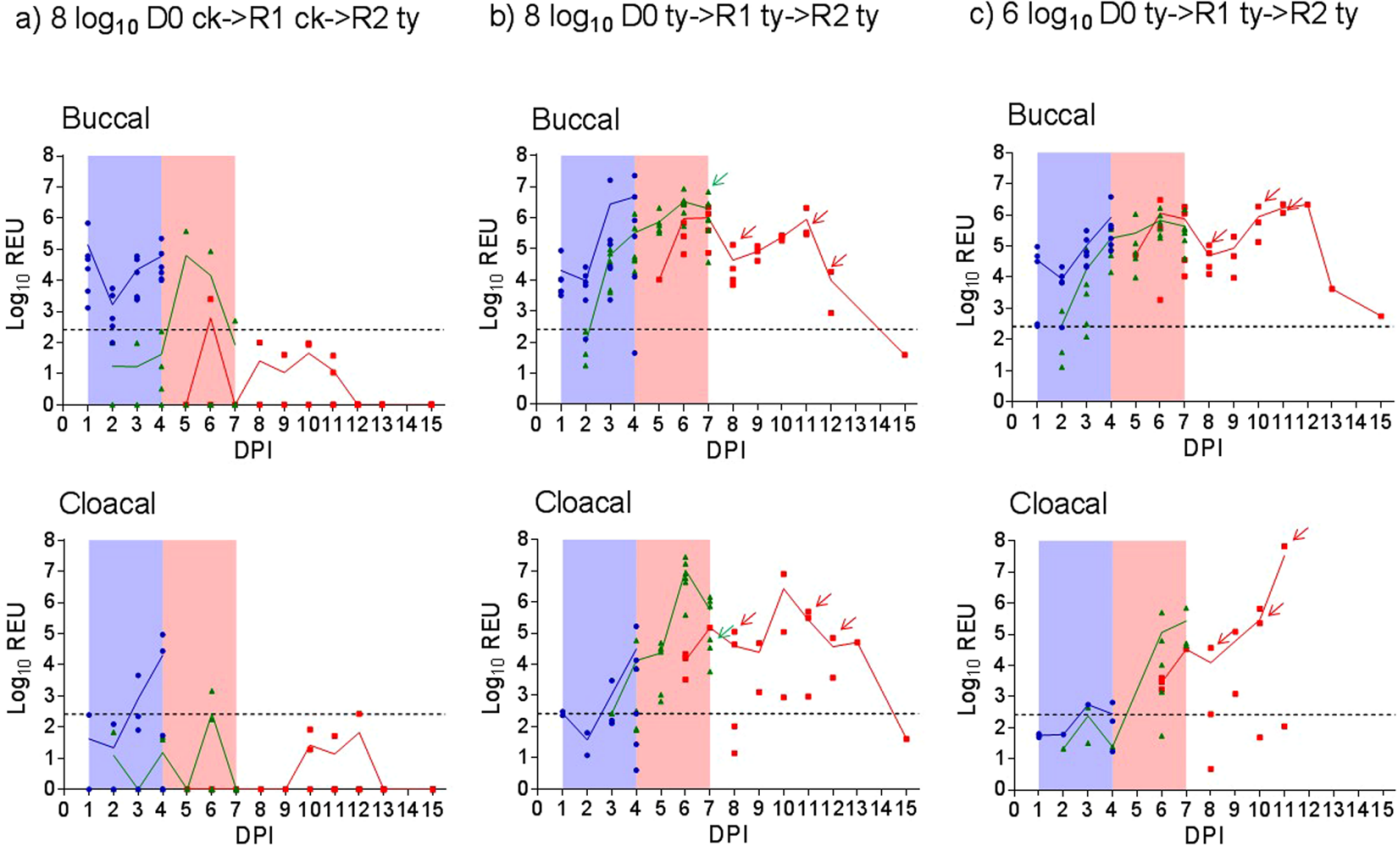
Buccal and cloacal H7N9 viral shedding (mean (line) and individual bird (symbols)) during direct- and contact-infection. (**a**,**b** and **c**) denote the three pens where doses of 8, 8 and 6 log_10_EID_50_ per bird were directly administered to the D0 group (n = 6) in each pen, namely chickens (ck), turkeys (ty) and turkeys respectively. R1 (n = 6) and R2 (n = 4) contact groups were introduced to each pen at 1 and 4 dpi respectively, in order to attempt the transmission: (**a**) D0 ck- > R1 ck- > R2 ty; (**b**) D0 ty- > R1 ty- > R2 ty and (**c**) D0 ty- > R1 ty- > R2 ty. Blue circles, green triangles and red squares indicate D0, R1 and R2 individual birds’ shedding titres respectively. See Fig. 1 and Methods section for planned withdrawl times of D0, R1 and R2 groups, with blue and pink shading indicating the D0/R1 and R1/R2 cohousing periods respectively. Arrows indicate final sampling of R1 and R2 turkeys in (b) and (c) which were either found dead or required euthanasia (Fig. 3). Broken horizontal line indicates the threshold for viral RNA REU shedding values.

Swabs and sera collected from all 60 birds prior to inoculation or exposure confirmed all to be negative for AIV shedding and seronegative by homologous HI and both anti-NP ELISAs. Direct-inoculation of D0 chickens and turkeys with the high 8log_10_ EID_50_ dose resulted in infection of all six (100%) in both pens (Fig. 2a and b, Supplementary Table S1). Introduction of R1 chickens and turkeys into the respective pens at 1 dpi for co-housing with the D0 birds of the same species (Fig. 1) resulted in two of six (33%) R1 chickens becoming infected (Fig. 2a, Supplementary Table S1). By contrast, all R1 turkeys (6/6; 100%) became infected (Fig. 2b). Replacement of D0 turkeys with R2 turkeys at 4 dpi (Fig. 1) resulted in onward transmission of infection to all four (100%) of the latter (Fig. 2b). Successful turkey transmission was observed in a third pen where the lower 6log_10_ EID_50_ dose also resulted in 6/6 (100%) D0, 6/6 (100%) R1 and 4/4 (100%) R2 turkeys becoming infected (Fig. 2c, Supplementary Table S1). Mean positive buccal shedding preceded cloacal shedding in D0 and R1 turkeys, but a similar time of mean shedding onset was observed for both tracts in the R2 turkeys (Fig. 2b and c). In a fourth pen, direct inoculation with the lowest (4log_10_ EID_50_) resulted in infection in 4/6 (67%) D0 turkeys with only 3/6 (50%) R1 turkeys infected, indicating reduced transmission at this inoculation dose (Supplementary Fig. S1 and Table S1). These experiments showed that >4log_10_ EID_50_ was the minimal infectious dose required to establish shedding in all D0 turkeys, with accompanying successful transmission of H7N9 infection to all R1 and then R2 contact turkeys as depicted in the schematic diagram (Fig. 1).

The ready susceptibility to contact infection in turkeys informed the decision to introduce four R2 turkeys at 4 dpi into the pen where chicken D0 to R1 transmission had been attempted and shown to be inefficient. Three of the four introduced R2 turkeys became infected (Fig. 2a and Supplementary Table S1), underlining the susceptibility of this species to contact infection. However, the mean shedding titres were generally lower and delayed in R1 and R2 contact turkey groups where infection was defined on the basis of single instances of positive shedding and/or sub-threshold shedding for two successive days (Fig. 2a, Supplementary Fig. S1 and Table S1).

### Mortality in contact-infected (R1 and R2) chickens and turkeys

**Figure 3:**
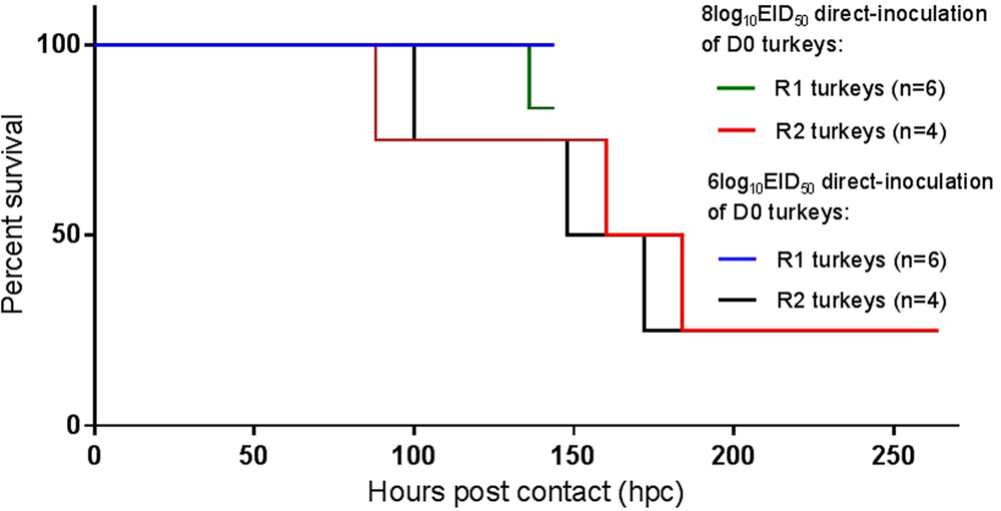
Kaplan-Meier survival plots of contact-infected R1 and R2 turkeys. Hours post contact denotes the time (hours) after introduction of the contact turkey groups into the two pens, i.e. at 4 and 7 dpi for the R1 and R2 contacts respectively (Fig. 1). The R1 turkeys were housed between 1–7 dpi, i.e. for a total of 144 hrs (6 days) maximum, with one death occurring in the 8log_10_EID_50_ pen at 136 hpc (5.67 dpc). The R2 turkeys were introduced at 96 hpc (4 dpi) and housed to the end of the study at 15 dpi, i.e. for 264 hrs maximum. However, R2 turkey deaths occurred between 100–172 hpc (mean death time (MDT): 140 hrs (5.83 days)) and 88–184 hpc (MDT: 144 hrs (6.0 days)) in the 6log_10_EID_50_ and 8log_10_EID_50_ pens respectively. No deaths occurred in any of the D0 turkeys directly-infected with either dose during their 4-day housing, i.e. prior to their removal at 4 dpi (not shown).

No mortality or overt clinical signs were observed in any of the D0 and R1 infected chickens. However, in the 8log_10_ EID_50_ turkey pen, 1/6 (17%) of R1 turkeys died suddenly at 136 hours post-contact (hpc (5.67 dpc)) with no obvious prior clinical signs (Fig. 3). A further 3/4 (75%) of the R2 turkeys in the same pen died between 88 and 184 hpc (mean time to death (MDT): 144 hpc (6.0 dpc)). In the 6log_10_ EID_50_ turkey pen there were no deaths among the six R1 infected turkeys, but 3/4 (75%) of the R2 turkeys died between 100 and 172 hpc (MDT: 140 hpc (5.83 dpc)) (Fig. 3). Five of the six R2 turkeys which died in both pens displayed ruffled feathers and huddled behaviour during the preceding day. Among these five R2 turkeys, one also had closed eyes and was incapable of independent feeding and drinking, while another developed drooping wings and an unsteady gait, hence both were euthanised. The sixth R2 turkey died suddenly, with no obvious clinical signs when inspected 1 hr earlier. The gross pathology of all seven turkeys which died (Fig. 3) was characterised by marked pancreatic haemorrhage (Supplementary Fig. S2). One apparently healthy R1 turkey which was pre-planned for cull at 6 dpc also displayed such gross haemorrhagic lesions of the pancreas (# 11, Table 1). There were no clinical signs nor deaths among any of the D0 and R1 infected turkeys in the pen where the initial inoculation dose was 4log_10_ EID_50_.

### Organ tropism of H7N9 LPAIV in chickens and turkeys

**Figure 4:**
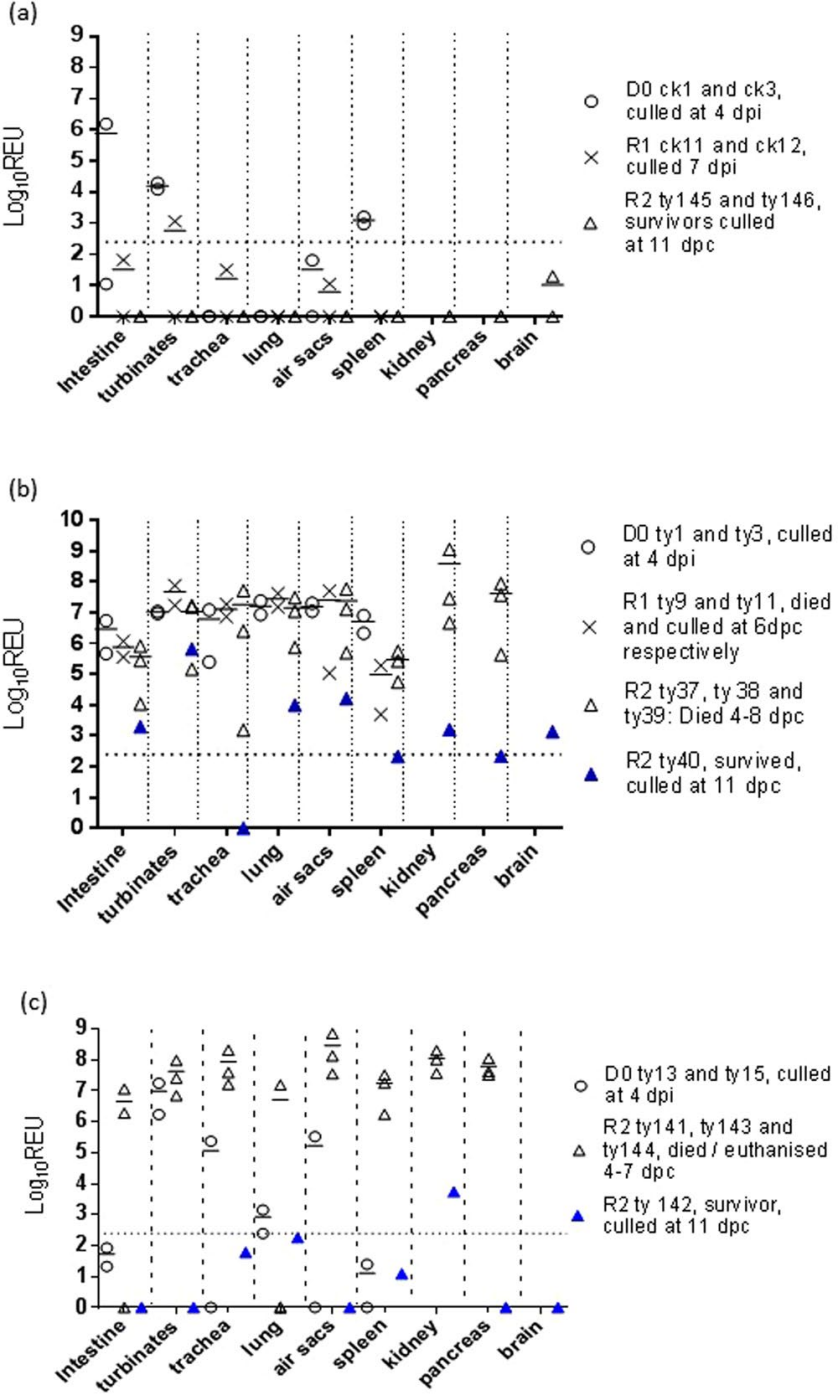
H7N9 viral RNA loads in organs obtained from infected birds. Horizontal bars indicate mean viral RNA loads when >1 bird was sampled from a given group at a given time/time window. Broken horizontal is the cut-off threshold, as explained in Fig. 2. (**a**) Log_10_ REU values in organs of D0 and R1 chickens (ck) plus R2 turkeys (ty) (8log_10_EID_50_ direct infection of D0 chickens); (**b**) Log_10_ REU values in organs of D0, R1 and R2 turkeys (8log_10_EID_50_ direct infection of D0 turkeys); (**c**) Log_10_ REU values in organs of D0 and R2 turkeys (6log_10_EID_50_ direct infection of D0 turkeys). Pancreas and kidney specimens were first collected for RNA extraction only when mortality became apparent in R2 turkeys, with the brain specimens collected only at R2 cull (11 dpc).

**Figure 5:**
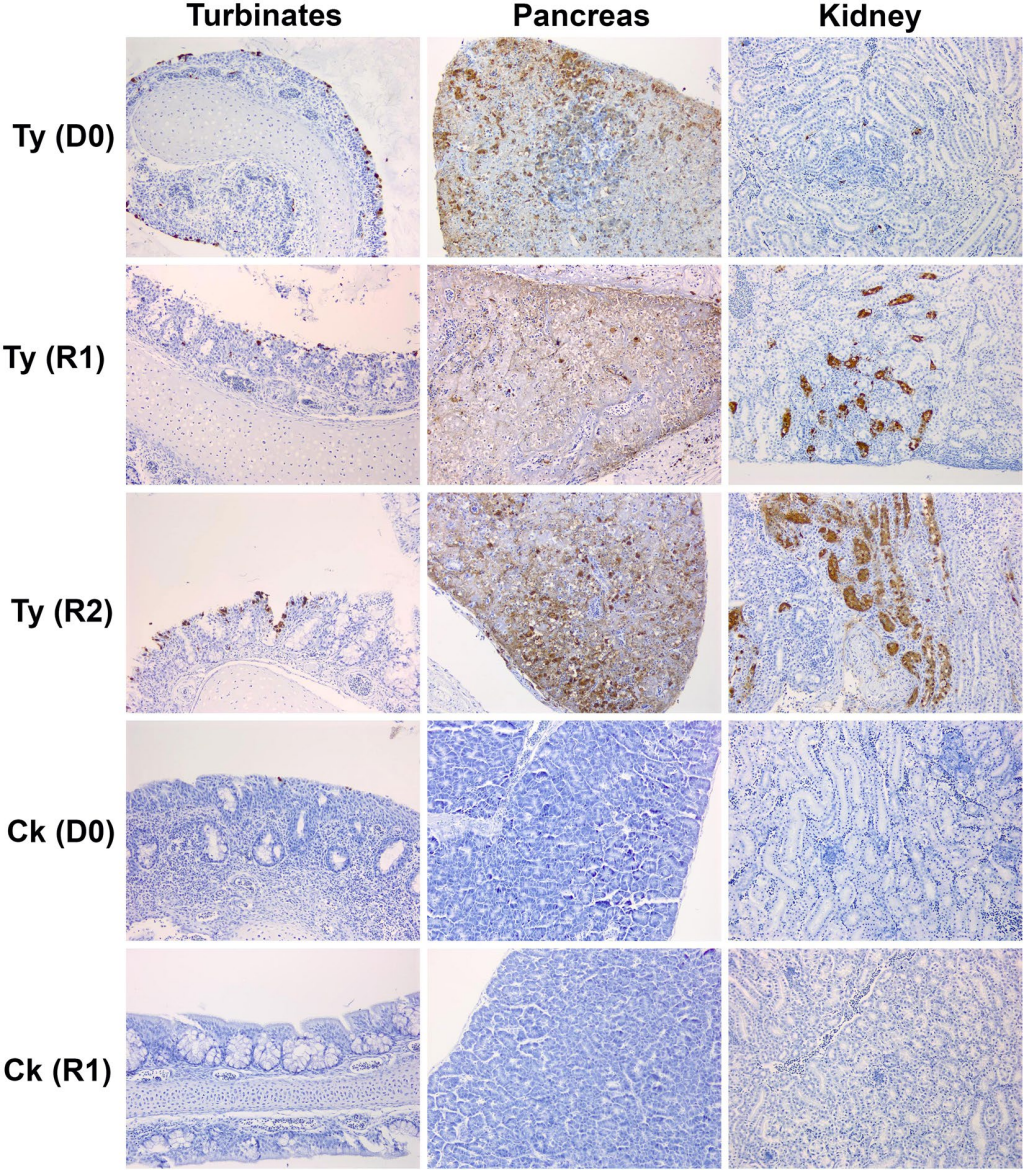
Examples of IAV-specific IHC staining. Demonstration of virus-specific immunolabelling (brown staining) of the NP in the turbinates, pancreas and kidney of turkeys and chickens where the initial D0 inoculation was 8log_10_ EID_50_ dose per bird. Organs from D0 and R1 birds were collected at 4 dpi (cull of both species) and 6 dpc (cull for chicken, death for turkey) respectively, with the R2 turkey sampled at death at 4 dpc. The R1 chicken was uninfected and its tissue sections served as a negative control for IHC.

H7N9 RNA was detected in the turbinates, intestine and spleen of D0 chickens, culled at 4 dpi (Fig. 4a). The IHC approach, known to be less sensitive than AIV RRT-PCR, revealed uncommon virus-specific staining in the turbinates (Fig. 5) of one D0 chicken and the caecal tonsil of another (Table 1). H7N9 viral RNA was also detected in the turbinates of one R1 infected chicken at cull, but all other organs were negative for H7N9 RNA (Fig. 4a).

In contrast to the D0 chickens, D0 turkeys directly-inoculated with the 8log_10_ EID_50_ dose harboured high H7N9 RNA titres in many organs when culled at 4 dpi, including the intestine, turbinates, trachea, lung, air sacs and spleen (Fig. 4b). IHC investigation affirmed systemic viral dissemination which included the pancreas, kidney, heart, liver and brain (Fig. 5 and Table 1). D0 turkeys which were infected with the lower 6log_10_ EID_50_ dose included several negative or sub-threshold viral RNA titres in organs (Fig. 4c). IHC also revealed less extensive or absent virus-specific staining in several organs compared to the D0 turkeys that had been infected with 8log_10_ EID_50_, thereby reflecting a dose/dissemination effect (Table 1). However, R1 and R2 turkeys in both pens harboured high viral titres in organs collected at time of cull or death (Fig. 4b and c), with IHC confirming systemic dissemination that included notable endothelial tropism (Table 1).

The two R2-infected turkeys which survived to the end of the experiment from the 8log_10_ and 6log_10_ EID_50_ pens were culled at 11 dpc (15 dpi) and had seroconverted by all four antibody tests (Supplementary Table S2). Both displayed a huddled stance and ruffled feathers for 2 days (turkey # 40) and 1day (turkey #142), commencing at 7 and 8 dpc respectively, followed by resolution. Organs sampled from both recovered R2 turkeys revealed reduced viral RNA titres compared those of R2 turkeys which died (Fig. 4b and c), and this was reflected in the absence of virus-specific IHC staining in both (Table 1 footnote). Examination of the cloacal shedding from these R2 recovered turkeys showed that both experienced a later onset and lower mean titres compared to the six R2 turkeys which had died (Supplementary Fig. S3).

### Seroconversion following H7N9 infection and/or exposure

D0, R1 and R2 infected birds were bled at cull, i.e. 4 dpi, 6 dpc and 11 dpc respectively. Progressive seroconversion to the homologous H7N9 HI antigen was observed (Supplementary Table S2). Among all the 31 available post-infection/exposure sera available for homologous H7 HI testing, 16/31 (52%) were seropositive, while the 32 sera tested by the anti-NP ELISAs included 10/32 (31%) which were seropositive by one of the two (i.e. the ID Vet or IDEXX AIV ELISA; n = 5) or both anti-NP ELISAs (n = 5). Only 4/32 (13%) of sera were H7 seropositive by the H7-specific ELISA.

### Genetic changes observed in H7N9 progeny viruses in infected birds

#### Molecular pathotyping of progeny viruses

The observation of unexpected mortality (75%) among the R2 turkeys (Fig. 3) indicated a distinct viral phenotype which contrasted with an absence of overt clinical signs in chickens. This difference prompted molecular pathotyping investigations of 52 swabs and one brain specimen from D0, R1 and R2 turkeys which all revealed an unchanged LPAIV CS sequence compared to the H7N9 wt inoculum, namely PEIPKGRGLF (Supplementary Table S3). The 10 chicken swabs obtained from D0 and R1 birds also revealed an unchanged LPAIV CS sequence.

#### L235Q polymorphisms in HA gene

**Figure 6:**
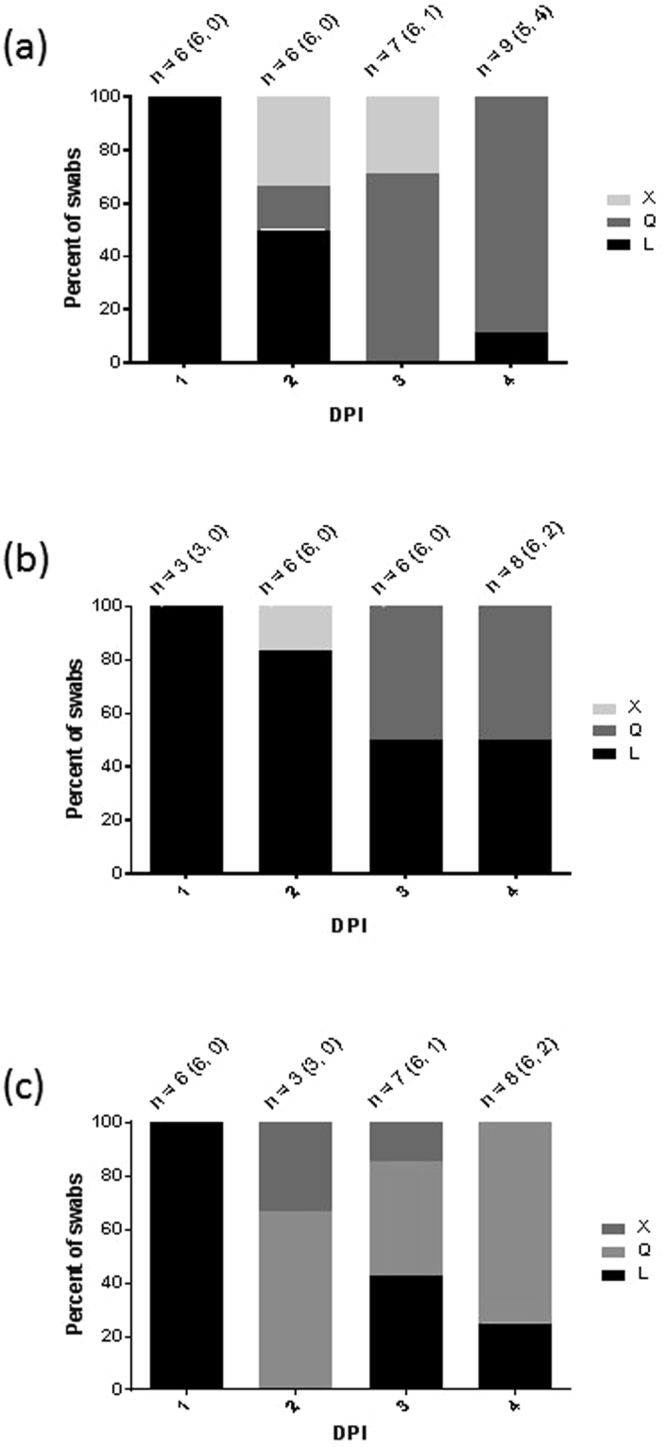
Changing proportions of polymorphisms at residue 235 in the viral HA gene of directly-inoculated (D0) birds swabbed at 1–4 dpi. (**a** and **b**) presents D0 turkeys inoculated with 8log_10_ and 6log_10_ EID_50_ of H7N9 wt respectively, while (**c**) presents D0 chickens inoculated with 8log_10_ EID_50_ of H7N9 wt. The total numbers of swabs of both types investigated at each daily time point are denoted by “n”, with the numbers of buccal and cloacal swabs distinguished in parenthesis (buccal, cloacal). All cloacal swabs contained Q235 except one in panel (**b**) at 4 dpi which contained L235.

Full-genome sequencing of the four R2 turkey and the one D0 chicken isolate all revealed the L235Q polymorphism to have occurred in the HA of all five (Table 2). Investigation of 51 swabs collected from D0 turkeys inoculated with the 8log_10_ and 6log_10_ EID_50_ doses showed that a mixed L/Q (X) polymorphism emerged followed by Q235 becoming increasingly prevalent by 4 dpi (Fig. 6a and b). All 85 (57 buccal, 28 cloacal) and all 37 swabs (24 buccal, 13 cloacal) sampled at 1–6 dpc and 1–8 dpc from R1 and R2 turkeys respectively possessed Q235, although two of the R1 turkey swabs were an X235 mixture. Investigation of 24 swabs from the infected D0 chickens displayed a very similar emergence of the X235 and Q235 polymorphisms (Fig. 6c). All four buccal swabs from the R1 chickens (sampled 3–6 dpc) possessed the Q235 polymorphism. No other amino acid changes were observed elsewhere in the HA genes of the four isolates obtained from the R2 turkeys and the D0 chicken (Table 2).

**Table 2:** Sequencing findings of H7N9 progeny viruses obtained as virus isolates from a directly-infected chicken (D0) and three infected contact turkeys (R2).

H7N9 dose for initial direct infection of donor (D0) birds	Identifier # for sampled bird (see Table 1)	Sampling of clinical specimens for VI at indicated time of cull or death (dpi or dpc), while * indicates sampling time of live ty	Unchanged H7 LPAIV CS confirmation by NGS of isolates	HA polymorphisms identified in HA by NGS of isolates of isolate	Amino acid changes in other (non-HA) genetic segments identified by NGS of isolates
8log_10_EID_50_	D0 ck (# 5)	4 dpi (culled), buccal swab	Yes	L235X	PA: N10H
8log_10_EID_50_	R2 ty (# 38)	7 dpc, kidney, FD (IVPI of turkey egg isolate: 0.39)	Yes	L235Q	Chicken egg isolate: NA: T10I; turkey egg isolate: NA: T10S
8log_10_EID_50_	R2 ty (# 40)	9 dpc*, cloacal swab, survived to study end at 11 dpc	Yes	L235Q	Chicken egg isolate: PB2: Y360H; turkey egg isolate: PB2: Y360H
6log_10_EID_50_	R2 ty (# 141)	7 dpc, cloacal swab, EUTH	Yes	L235Q	No changes in other segments
6log_10_EID_50_	R2 ty (# 141)	7 dpc, pancreas, EUTH	Yes	L235Q	Chicken egg isolate: PA: R269K; turkey egg isolate: PA: R269K, PB1-F2: M1T

Full-genome sequences were obtained by NGS of the D0 chicken H7N9 isolate (obtained by VI in chicken eggs), while both chicken and turkey eggs were used to isolate H7N9 from the R2 turkeys. Further evidence for an unchanged LPAIV CS during H7N9 *in vivo* infection and transmission is summarised in Supplementary Table S3. The broader occurrence of the identified polymorphisms in the PB2, PB1-F2, PA and NA genes was investigated by analysing all available China-origin H7N9 gene sequences (Supplementary Table S4). Species abbreviations: ck = chicken; ty = turkey; FD = found dead; EUTH = euthanised; NGS = Next generation sequencing.

#### Changes in other non-HA genetic segments

Additional amino acid changes were identified in other gene segments among three of the four isolates obtained from the R2 turkeys and the D0 chicken, although for the turkey-derived isolates there were some differences depending on whether chicken or turkey eggs were used for isolation (Table 2). These polymorphisms were identified as changes relative to the H7N9 wt inoculum, with the PA R269K, NA T10I/S and PB1-F2 M1T (possible ablation of peptide) changes having also occurred in other sequenced China-origin H7N9 isolates (Supplementary Table S4). However, the PB2 Y360H and PA N10H polymorphisms appear to be unique to the viral progeny obtained in the current study.

### IVPI

IVPI tests were done on two H7N9 LPAIV isolates, namely the EP6 inoculum stock (H7N9 wt, chicken egg passaged) and the isolate recovered in turkey eggs from the kidney of a R2 turkey which died, referred to as H7N9 turkey-adapted (ty-adapted) (Table 2). Both the H7N9 wt and ty-adapted isolates registered IVPI scores of 0.00 and 0.39 respectively which were <1.2, therefore confirming the officially-defined LPAIV phenotype for both.

## Discussion

While H7N9 LPAIV infection of chickens caused no clinical signs as in previous studies^14–19^, unexpected mortality occurred in 75% of infected R2 turkeys (Fig. 3) following efficient transmission (Fig. 2b and c, Supplementary Table S1). Virulent infection resembled HPAIV pathogenesis and mortality, with H7N9 detection in feather follicles of D0-infected turkeys (Table 1) implying that viremia may have systemically disseminated the infection, reflecting similarities with viral tropism in feathers during H5N1 HPAIV outbreaks^36,37^. Examination of turkey brain tissues revealed infection (Table 1 and Fig. 4b), thereby also resembling HPAIV tropism^38^. However, investigation of the CS sequences in the turkey progeny viruses revealed the LPAIV CS (monobasic) sequence to be unchanged throughout transmission (Supplementary Table S3), with the LPAIV phenotype (IVPI) confirmed by testing an R2 turkey isolate, namely H7N9 ty-adapted. It was important to establish that no mutation had occurred in the monobasic LPAIV CS during transmission in turkeys, particularly in view of the emergence of H7N9 HPAIV variants during the “fifth wave” in China during winter 2016–2017^12,13^. Inefficient transmission from D0 to R1 chickens reflected other studies which described considerable variability in H7N9 LPAIV transmission within this host^14–16,18,19^.

Little or no mortality is normally observed during most H7 LPAIV turkey outbreaks^39–41^, but up to 40% mortality has been reported in association with secondary bacterial infections and husbandry factors^42,43^. Experimental infection of high health status turkeys with H7 LPAIVs resulted in up to 40–60% mortality for three of 12 North American isolates^44^, but without the extensive systemic dissemination described in the current study (Figs 4 and 5, Table 1). Because SPF turkeys are unavailable, the H7N9 LPAIV study also used high health status turkeys which were unlikely to be complicated by secondary bacterial infections, with the absence of exudate in the lungs and no Gram staining in tissue sections (data not shown) indicating that secondary infection was unlikely to have contributed to the H7N9 LPAIV virulence. The containment facilities included air supply via HEPA filters which reduced the risk of acquiring exogenous respiratory pathogens. Extensive endothelial tropism was clear, and in a number of organs infection had also spread into the adjacent parenchymal tissues (Table 1), hence this widespread pathogenesis is likely to have contributed to multiple-organ failure in turkeys^45^, as exemplified by gross haemorrhage in the pancreas (Supplementary Fig. S2).

Possible reasons for the unusual virulence in turkeys merit consideration. IAV host adaptation and pathogenesis mechanisms are emphasised in pandemic concerns regarding avian to human adaptation^46–48^, but the context of this avian study differed in that clearly distinct outcomes resulted in two poultry hosts. The L235Q change in the HA was detected soon after infection of D0 birds (both species) and was maintained during efficient transmission to R2 turkeys. This polymorphism is long-known to affect avian to mammalian adaptation of IAVs, whereby Q235L influences a general change in host cell receptor preference from those which possess α-2,3 to α-2,6 sialic acid linkages, known as “avian” and “human” receptors respectively, although H7N9 wt (L235) binds to both α-2,3 and α-2,6 receptors with a slight preference for the former^49^. The L235Q polymorphism appears to be a reversion away from human receptor binding^50^, and was previously reported following chicken infection both in *in vivo* and *in ovo*^17^. It is speculated that the distribution of α-2,3 receptors in turkeys may enable the greater H7N9 dissemination within turkeys compared to chickens and was supported by the four D0 turkeys sacrificed at 4 dpi which displayed differing organ tropism: The two which received 8log_10_ EID_50_ were already experiencing extensive systemic distribution while the two which received 6log_10_ EID_50_ displayed typical LPAIV turkey tropism largely restricted to the respiratory tract^51^, with the former two having already changed to Q235 while the latter two turkeys possessed L235 (Table 1). A dose effect is the likely cause of these differences, but it is speculated that a greater distribution of avian receptors in multiple turkey tissues may have assisted systemic dissemination of LPAIV infection (with consequences for virulence) following the L235Q switch. Both receptor types broadly occur in the turkey respiratory and enteric tracts^52,53^, but their distribution in other organs remains to be investigated.

Polymorphisms at other residues in the HA are also known to influence its acid-stability which is the optimal pH at which HA-driven fusion of the viral envelope occurs with the endosomal membrane at the early stages of intracellular infection^54^. The fusion pH correlates with phenotypic changes that include the IAV avian to mammalian adaptation switch. For example, HA acid-stability was altered by mutating particular residues in H5N1 HPAIV which changed its transmission and pathogenesis among ducks^55^, which also affected these phenotypic characteristics in mice and ferrets (reviewed by Russell^54^). In a purely avian context, the fusion pH of two closely related H7N3 LPAIVs was studied using a mallard-derived (precursor) isolate and its derivative which caused turkey outbreaks. The two isolates differed in the HA and NA at one positon in each, with the turkey isolate experiencing viral/endosomal fusion at a higher pH than the mallard isolate which may reflect adaptation to the avian host species of origin^56^. However, other than the L235Q switch, the HA protein sequences of the four H7N9 R2 turkey isolates and the chicken D0 isolate remained identical (Table 2), suggesting that HA acid-stability may be an unlikely factor in explaining differences in transmission, systemic dissemination and pathogenesis in both hosts. The fusion pH in H7N9 LPAIV decreased due to the L235Q polymorphism^50^, but it is unclear whether this altered acid-stability may influence the enhanced dissemination and virulence in turkeys.

Interestingly, acid-stability changes correlated with altered sensitivity of different IAVs (including a reverse-genetics H7N9) to anti-viral host responses which included interferon-induced transmembrane proteins which inhibit viral and cellular membrane fusion^57^. More broadly, it is possible that differences between chicken and turkey host responses to H7N9 LPAIV infection may influence the distinct organ tropism and pathogenesis. The possible role of an immune response was underlined by the two R2 turkeys which resolved infection and survived to the end of the study. Both turkeys experienced a delayed onset and generally lower titres of enteric shedding compared to the R2 turkeys which died (Supplementary Fig. S3). It is speculated that the two turkeys produced a successful host response to limit viral replication and tropism, these observations being more akin to a typical LPAIV infection in this species^51^, while the turkeys which died did not. *In vitro* studies have shown that innate immune and apoptotic responses differ in chickens and ducks following H5N1 HPAIV infection which may in turn affect the clear differences in clinical outcomes in these two species^58,59^. Infection of chickens, quails and ducks with H7N9 LPAIV resulted in the strongest early IFN-α and TLR-7 responses occurring in the chickens, with the authors noting that this antiviral response correlated with lower viral replication in this species compared to quail^19^. However, there is presently limited knowledge concerning further details of turkey host responses to any AIV infection, particularly the role of innate immunity.

Endothelial tropism in many organs which distinguished H7N9 LPAIV infection of turkeys from chickens has already been noted (Table 1), with a recent study showing that innate immunity escape may enable this localisation for some LPAIV strains which may contribute to severe LPAIV infections in galliforme poultry^60^. LPAIV tropism in avians is classically restricted to the respiratory and enteric tracts due to the localisation of trypsin as the protease required for HA CS activation during infection^47^. However, chicken H9N2 *in vitro* studies have shown that other proteases may activate the LPAIV CS^61,62^, and in some circumstances include the ubiquitous furin protease, normally associated with HPAIV CS activation^63^. While there are no reported descriptions of alternative proteases in turkey tissues, it is speculated that non-trypsin proteases may have facilitated H7N9 LPAIV CS activation in multiple visceral organs.

Intravenous inoculation of both H7N9 variants in chickens yielded a higher IVPI score for ty-adapted compared to wt because of one chicken mortality. The four full-genome sequenced R2 progeny viruses from turkeys and the chicken-derived isolate included polymorphisms in the non-HA genetic segments (Table 2), but there was no clear association between these and any known pathogenicity markers, with some of the polymorphisms having been previously observed in China-origin H7N9 (both LPAIVs and HPAIVs) since 2013 (Table S4). Further infection studies with both H7N9 LPAIV wt and ty-adapted variants in turkeys (including intravenous administration, albeit this is not a natural route of infection) may provide clues concerning pathogenesis mechanisms in this host.

In view of the continuing viral evolution and expansion of infection in Chinese poultry, these novel findings will contribute to continuing risk assessments concerning future spread of this H7N9 LPAIV beyond China^21,22^. Serological approaches will remain important for active surveillance in chickens^64^, while, unusually for LPAIVs, passive surveillance may be also required to monitor for China-origin H7N9 LPAIV incursions into turkeys. In conclusion, this study presented a potentially dangerous notifiable disease scenario where, in the event of China-origin H7N9 LPAIV spreading beyond Asia, undetected maintenance within the chicken sector may be followed by introduction, spread and significant mortality in turkeys.

## Electronic supplementary material

Supplementary Information
