# Supplementary material for: Unexpected infection outcomes of China-origin H7N9 low pathogenicity avian influenza virus in turkeys
Source: Sci Rep. 2018 May 9;8:7322. doi: 10.1038/s41598-018-25062-y (PMC5943237; doi:10.1038/s41598-018-25062-y)
Supplement: Supplementary file 1 — Supplementary Information [file 41598_2018_25062_MOESM1_ESM.doc]

**Supplementary figures and tables**

**Unexpected infection outcomes of China-origin H7N9 low pathogenicity**

**avian influenza virus in turkeys**

Marek J Slomka*,^1^ Amanda H Seekings,^1^ Sahar Mahmood,^1^ Saumya Thomas,^1^ Anita Puranik, ^1^ Samantha Watson,^2^ Alexander MP Byrne,^1^ Daniel Hicks,^3^ Alejandro Nunez,^3^ Ian H Brown,^1^ Sharon M Brookes.^1^

Virology Department,^1^ Animal Services Unit ^2^ and Pathology Department,^3^ Animal and Plant Health Agency (APHA-Weybridge), Woodham Lane, Addlestone, Surrey KT13 3NB, United Kingdom.

*corresponding author: [marek.slomka@apha.gsi.gov.uk](mailto:marek.slomka@apha.gsi.gov.uk)

Figure S1

Buccal shedding

Cloacal shedding

**Figure S1. H7N9 viral shedding in D0 and R1 turkeys following direct-inoculation with 4 log_10_EID_50_.** Top and bottom panels indicate shedding titres (mean and from individual birds) from buccal and cloacal cavities respectively. Blue background colour indicates 3-day cohousing period of D0 and R1 turkeys, the former being withdrawn at 4 dpi, while the later was introduced at 1 dpi and with drawn at 7 dpi (i.e. 6 dpc). No mortality occurred in any of the D0 and R1 turkeys. Broken horizontal line indicates the threshold cut-off for REU shedding values (see main text, “Materials and Methods” section).

Figure S2


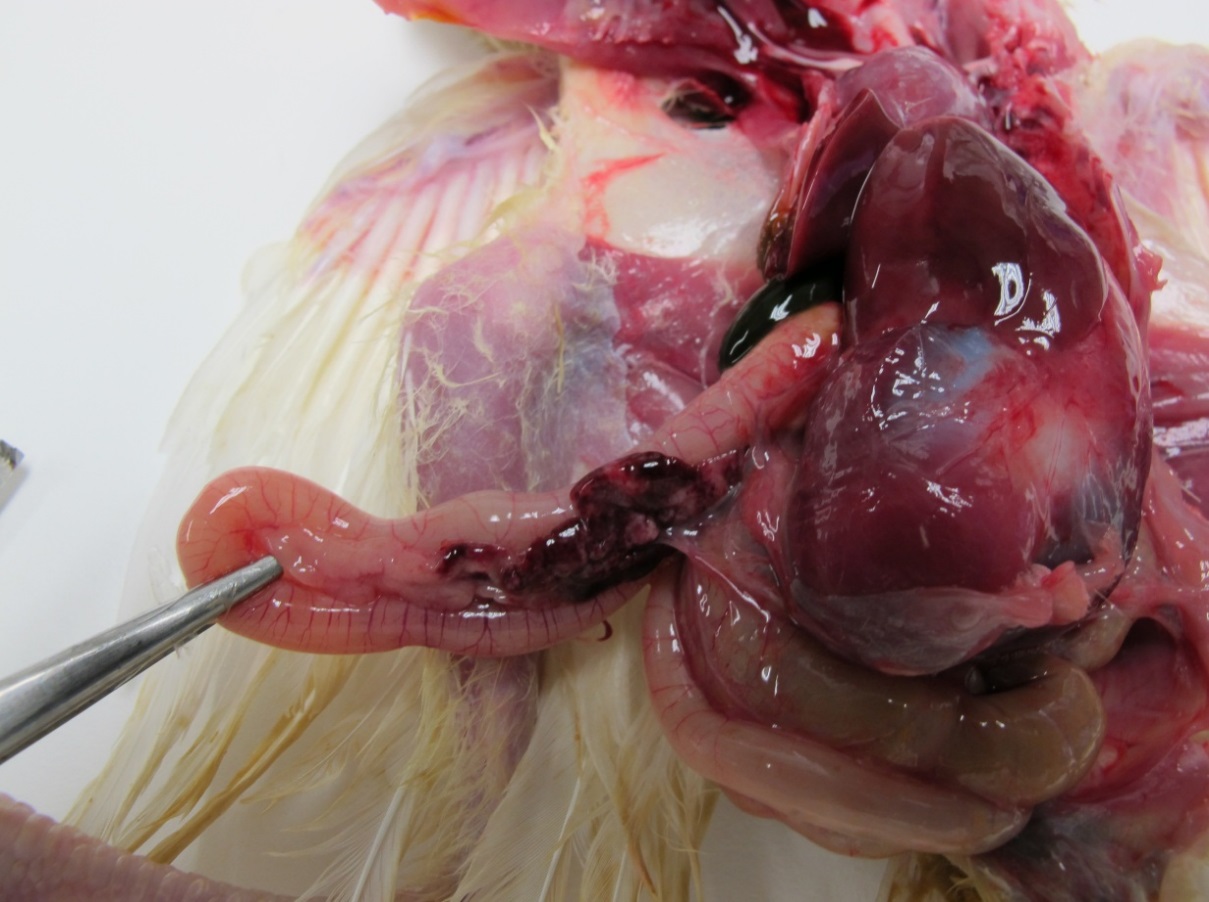


**Figure S2. Pancreas: Gross pathology at time of mortality.** Arrow indicates haemorrhage on part of pancreas from an R2 turkey which was found dead at 7 dpc (# 38, Table 1).

Figure S3

**Figure S3. Comparison of mean cloacal shedding in R2 turkeys which died (n=6) and survived (n=2).** These eight R2 turkeys were swabbed in the pens where initial direct-inoculation of D0 turkeys was with 8 log_10_EID_50_ and 6 log_10_EID_50_ of H7N9 wt. Mean cloacal shedding is indicated by unbroken lines for 1-11 dpc which corresponds to 5-15 dpi, while individual cloacal shedding from the six turkeys which died and the two which survived are indicated by filled squares and open circles respectively.

Table S1

| **Pen** | | **Proportions of directly-infected or contact birds** | | |
| --- | --- | --- | --- | --- |
| **Inoculation dose of D0 birds** | **Transmission chain (see corresponding figure)** | **D0**  **(n=6)** | **R1**  **(n=6)** | **R2**  **(n=4)** |
| 8log10EID50 | Ck-Ck-Ty (Fig. 2A) | 6/6 | 2/6 (*0/4*) | 2*/4 (*1/2*) |
| 8log10EID50 | Ty-Ty-Ty (Fig. 2B) | 6/6 | 6/6 | 4/4 |
| 6log10EID50 | Ty-Ty-Ty (Fig. 2C) | 6/6 | 6/6 | 4/4 |
| 4log10EID50 | Ty-Ty (Fig. S1) | 2/6 (*2/4*) | 3/6 (*0/3*) |  |

**Table S1. Proportions of H7N9 LPAIV infected birds in each of the four pens.** Classification of infection is indicated by:

1. Numbers of birds in normal type which experienced at least one instance of positive shedding throughout sampling, with * indicating two R2 infected turkeys which each included only one instance of positive shedding and were shown to have seroconverted by 11 dpi (see HI results for ty # 145 (1:256) and 148 (1:16), Supplementary Table S2).
2. Italic numbers (in parenthesis) which denote birds which did not include any instance of positive shedding but experienced sub-threshold shedding on two consecutive days. This criterion identified three additional infected turkeys. See supplemental Table S2 for partial (1:8, drawn at 4 dpi) and positive (1:64, drawn at 11 dpc) HI seroconversion data which verified infection for ty # 27 and # 147 respectively, but no serum was available for the third infected turkey which similarly shed at sub-threshold levels for two successive days.

Ck = chicken, Ty = turkey.

Table S2

| **Sampling time at cull** | **Infection route** | **Direct-infection dose details for a given group (pen) of D0 turkeys and D0 chickens*** | **Bird ID #** | **Homologous HI H7N9 Anhui/1/13** | | | **ELISA Results** | | |
| --- | --- | --- | --- | --- | --- | --- | --- | --- | --- |
|  |  |  |  | **Proportions of HI positives** | **Individual titres** | **Geometric mean titres per group (two or more sera)** | **IDEXX AIV generic (NP)** | **IDVET AIV generic (NP)** | **IDVET H7-specific** |
| **4 dpi** | Directly Infected (D0) | 8log10EID50 | **Ty 5** | 0/2 | 8 | 8 | + ^ | - | - |
|  |  |  | **Ty 6** |  | 8 |  | + ^ | - | - |
|  |  | 6log10EID50 | **Ty 16** | 0/2 | 8 | 8 | - | - | - |
|  |  |  | **Ty 18** |  | 8 |  | - | - | - |
|  |  | 4log10EID50 | **Ty 26** | 0/2 | 8 | 8 | - | - | - |
|  |  |  | **Ty 27** |  | 8 |  | - | - | - |
|  |  | 8log10EID50* | **Ck 2** | 0/1 | 8 |  | - | - | - |
| **7 dpi (6 dpc)** | Contacts (R1) | 8log10EID50 | **Ty 7** | 3/3 | 256 | 512 | - | + | - |
|  |  |  | **Ty 8** |  | 1024 |  | + | + | Borderline |
|  |  |  | **Ty 10** |  | 512 |  | + | + | - |
|  |  | 6log10EID50 | **Ty 19** | 3/5 | 8 | 24.3 | - | - | - |
|  |  |  | **Ty 20** |  | Insufficient serum available |  | - | - | - |
|  |  |  | **Ty 21** |  | 16 |  | - | - | - |
|  |  |  | **Ty 22** |  | 64 |  | - | - | - |
|  |  |  | **Ty 23** |  | 8 |  | - | + | - |
|  |  |  | **Ty 24** |  | 128 |  | + | + | - |
|  |  | 4log10EID50 | **Ty 31** | 1/3 | 32 | 12.7 | - | - | - |
|  |  |  | **Ty 33** |  | 8 |  | - | + | - |
|  |  |  | **Ty 36** |  | 8 |  | - | - | - |
|  |  |  | ***Ty 32*** | *1/3* | *8* | *8* | *-* | *-* | *-* |
|  |  |  | ***Ty 34*** |  | *4* |  | *-* | *-* | *-* |
|  |  |  | ***Ty 35*** |  | *16* |  | *-* | *-* | *-* |
|  |  | 8log10EID50* | **Ck 7** | 1/1 | 256 |  | - | - | - |
|  |  |  | ***Ck 8*** | 1/3 | *32* | *10.1* | - | - | - |
|  |  |  | ***Ck 9*** |  | *4* |  | - | - | - |
|  |  |  | ***Ck 10*** |  | *8* |  | - | - | - |
| **15 dpi (11 dpc)** | Contacts (R2) | 8log10EID50 | **Ty 40** | 1/1 | 512 |  | + | + | + |
|  |  | 6log10EID50 | **Ty 142** | 1/1 | 512 |  | + | + | + |
|  |  | 8log10EID50* | **Ty 145** | 3/3 | 256 | 64 | - | - | - |
|  |  |  | **Ty 147** |  | 64 |  | - | - | - |
|  |  |  | **Ty 148** |  | 16 |  | - | - | - |
|  |  |  | ***Ty 146*** | *1/1* | *128* |  | *-* | *-* | *Borderline* |
| **Proportion of total sera (%) which were positive by stated test:** | | | | 16/31 (52%) | | | 7/32 (22%) | 8/32 (25%) | 4/32 (13%) which includes the two borderline results as positive |

**Table S2. Serology results for sera from 32 birds, namely 27 turkeys (ty) and five chickens (ck), sampled at cull.** Red numerals and symbols indicate positive results for both HI (titres shown as reciprocals) and ELISA tests. Asterisk * indicates sera drawn from the ck-ck-ty transmisson chain (Fig. 2A). Italic HI titres and text indicates seven contact birds (four turkeys and three chickens) which were not infected (as defined in the main text), yet were seropositive (i.e. reciprocal HI titre equal or >16 in three of these birds) or possibly seroconverting (reciprocal HI titres of 4 and 8 in four of these birds) by homologous HI, but all seven were seronegative by both anti-NP ELISAs. These results may indicate that these seven birds experienced very limited viral relication where positive shedding was suppressed by a successful immune response which led to HI seroconversion / possible ongoing seroconversion by 6 and 11 dpc. ^ The IDEXX ELISA registered positive anti-NP titres for two sera which were drawn at 4 dpi: It was speculated that these two early anti-NP positive results may be a consequence of a relatively rapid immunological response to a high dose of inoculum which was administered directly.

Table S3

| **Species** | **Dose administered to D0 birds** | **Numbers of swabs tested (buccal, cloacal)** | | | | Total number of swabs tested for unchanged LPAIV CS per pen |
| --- | --- | --- | --- | --- | --- | --- |
|  |  | **D0** | **R1** | **R2 dead turkeys** | **R2 survivor turkeys** |  |
| Turkey | 8log_10_ EID_50_ | 9 (5, 4) | 12* (6, 6) | 6 (3, 3) | 1 ^ (0, 1) | 28 swabs (^ plus one brain) from  16 turkeys |
| Turkey | 6log_10_ EID_50_ | 8 (6, 2) | 9 (6, 3) | 6 (3, 3) | 1 (1, 0) | 24 swabs from  16 turkeys |
| Chicken | 8log_10_ EID_50_ | 8 (6, 2) | 2 (2, 0) | CS not investigated in the R2 turkey swabs in this pen | | 10 swabs from seven chickens |

**Table S3. Unchanged LPAIV cleavage site (CS) detected in 52 turkey swabs and 10 chicken swabs.** The table summarises the sources of the swabs where amplicons were successfully sequenced after initial amplification of extracted RNA by the H7 CS conventional RT-PCR. The swabs (buccal and cloacal) were obtained from 32 turkeys and seven chickens at different stages of the H7N9 LPAIV transmission chain. The D0 and R1 birds were culled at 4 dpi and 6 dpc respectively, with at least one successful amplicon obtained from all six turkeys in each group, with * indicating a pair of swabs from one “found dead” turkey at 6 dpc. At least one amplicon was obtained from the six D0 chickens and one R1 chicken. The six R2 turkeys died between 4-8 dpc, with three carcasses from each pen yielding six successful amplifications for sequencing. Amplified swabs were obtained at 8 and 9 dpc from the two R2 turkeys which survived to the end of the study at 11 dpc, at which point the surviving turkey from the 8log_10_ EID_50_ pen provided ^ an additional brain tissue which was similarly tested to also reveal an unchanged LPAIV CS. The 62 swabs and brain specimen were of sufficiently high H7N9 viral load (i.e. mainly Ct values of approximately <30-32) to successfully amplify by the H7 CS conventional RT-PCR.

Table S4

| **Gene** | **Polymorphism detected in H7N9 LPAIV progeny in current study (Table 2)** | **Number of China-origin H7N9 sequences checked** | **Previously observed China-origin H7N9 polymorphism at the same position in a given gene, with *italic type referring to the corresponding polymorphism observed in the current study*** | **H7N9 pathotype** |
| --- | --- | --- | --- | --- |
| PB2 | Y360H | 1568 | Majority are Y except for:  **Y360F:**  A/Guangxi/18895/2017 EPI_ISL_268503  **Y360C:**  A/chicken/Jiangxi/10945/2014_EPI_ISL_176116  *Y360H change in current study appears to be unique for China-origin H7N9* | HP  LP |
| PB1-F2 | M1T | 1562 | Majority are M except for:  **M1T:**  A/Qingyuan/GIRD1/2017_EPI_ISL_249102  A/Hunan/02287/2017 EPI_ISL_242845  A/Guangdong/17SF006/2017 EPI_ISL_249308  A/Qingyuan/GIRD01/2017 EPI_ISL_257022  *PB1-F2 ablation observed previously in four instances, including two H7N9 HPAIVs* | LP  LP  HP  HP |
| PA | N10H | 1471 | All are N  *N10H change in current study appears to be unique for China-origin H7N9* |  |
| PA | R269K | 1568 | Majority are R except for:  **R269K**:  A/duck/Hunan/S11682/2015_EPI_ISL_283538  A/duck/Shanghai/SD016/2015_EPI_ISL_283545  A/duck/Zhejiang/LS02/2014_EPI_ISL_174489  A/Fujian/16/2014_EPI_ISL_192341  A/pigeon/Zhejiang/P2/2013_EPI_ISL_162875  A/Zhejiang/4/2017_EPI_ISL_242851  **R269I:**  A/GD-51/2015/H7N9/2015-01-30_EPI_ISL_198737  A/GD-68/2015/H7N9/2015-02-08_EPI_ISL_198742  A/Guangdong/15SF051/2015_EPI_ISL_192283  A/Hong_Kong/5581/2014_EPI_ISL_159477  *R269K change observed previously in six H7N9 LPAIV instances* | LP  LP  LP  LP  LP  LP  LP  LP  LP  LP |
| NA | T10I/S | 1623 | Majority are T except for:  **T10S:**  A/duck/Zhejiang/S4488/2014_EPI_ISL_283547  A/duck/Shanghai/SD016/2015_EPI_ISL_283545  A/duck/Shanghai/SD015/2015_EPI_ISL_283544  A/Anhui/DEWH72-02/2013_EPI_ISL_188972  **T10I:**  A/chicken/Zhejiang/S4017/2013_EPI_ISL_283529  A/Anhui/01/2013-CDC-LV7A_EPI_ISL_159416  A/Anhui/DEWH72-06/2013_EPI_ISL_188976  A/Anhui/DEWH72-07/2013_EPI_ISL_188977  A/Anhui/DEWH72-02/2013_EPI_ISL_188972  **T10A**:  A/Guangxi/18906/2017_EPI_ISL_268506  *T10I/S change observed previously in nine H7N9 LPAIV instances* | LP  LP  LP  LP  LP  LP  LP  LP  LP  HP |

**Table S4.** **Non-HA genetic polymorphisms identified in the H7N9 LPAIV progeny isolates and their occurrence in other China-origin H7N9 sequences**. Four viral isolates were obtained from R2 turkeys and one from a D0 chicken in the current study which revealed the polymorphisms listed in Table 2.
